# Automated MRI perfusion-diffusion mismatch estimation may be significantly different in individual patients when using different software packages

**DOI:** 10.1007/s00330-020-07150-8

**Published:** 2020-08-21

**Authors:** Hannes Deutschmann, Nicole Hinteregger, Ulrike Wießpeiner, Markus Kneihsl, Simon Fandler-Höfler, Manuela Michenthaler, Christian Enzinger, Eva Hassler, Stefan Leber, Gernot Reishofer

**Affiliations:** 1 Department of Radiology, Division of Neuroradiology, Vascular and Interventional Radiology, Medical University of Graz, Auenbruggerplatz 9, 8036 Graz, Austria; 2 Department of Neurology, Division of General Neurology, Medical University of Graz, Auenbruggerplatz 22, 8036 Graz, Austria

**Keywords:** Brain, Thrombectomy, Stroke, Magnetic resonance imaging, Software

## Abstract

**Objective:**

To compare two established software applications in terms of apparent diffusion coefficient (ADC) lesion volumes, volume of critically hypoperfused brain tissue, and calculated volumes of perfusion-diffusion mismatch in brain MRI of patients with acute ischemic stroke.

**Methods:**

Brain MRI examinations of 81 patients with acute stroke due to large vessel occlusion of the anterior circulation were analyzed. The volume of hypoperfused brain tissue, ADC volume, and the volume of perfusion-diffusion mismatch were calculated automatically with two different software packages. The calculated parameters were compared quantitatively using formal statistics.

**Results:**

Significant difference was found for the volume of hypoperfused tissue (median 91.0 ml vs. 102.2 ml; *p* < 0.05) and the ADC volume (median 30.0 ml vs. 23.9 ml; *p* < 0.05) between different software packages. The volume of the perfusion-diffusion mismatch differed significantly (median 47.0 ml vs. 67.2 ml; *p* < 0.05). Evaluation of the results on a single-subject basis revealed a mean absolute difference of 20.5 ml for hypoperfused tissue, 10.8 ml for ADC volumes, and 27.6 ml for mismatch volumes, respectively. Application of the DEFUSE 3 threshold of 70 ml infarction core would have resulted in dissenting treatment decisions in 6/81 (7.4%) patients.

**Conclusion:**

Volume segmentation in different software products may lead to significantly different results in the individual patient and may thus seriously influence the decision for or against mechanical thrombectomy.

**Key Points:**

*• Automated calculation of MRI perfusion-diffusion mismatch helps clinicians to apply inclusion and exclusion criteria derived from randomized trials.*

*• Infarct volume segmentation plays a crucial role and lead to significantly different result for different computer programs.*

*• Perfusion-diffusion mismatch estimation from different computer programs may influence the decision for or against mechanical thrombectomy.*

## Introduction

Mechanical thrombectomy (MT) performed within 6 h from symptom onset is regarded as gold standard therapy in patients with acute ischemic stroke caused by large vessel occlusion (LVO) in the anterior circulation [1–9]. Recently, two prospectively randomized trials demonstrated the value of MT in selected patients with a prolonged time interval from symptom onset or the time last seen well and MT, i.e., 6 to 16 h in DEFUSE3 [10] and 6 to 24 h in DAWN [11]. Therefore, following the current guidelines from the American Heart Association/American Stroke Association (AHA/ASA) for acute stroke treatment, MR- or CT-based perfusion and MR diffusion imaging is advised in an extended time window up to 24 h [12]. In the light of these findings, more refined knowledge of the performance of user-independent quantification of the infarct volume and/or the volume of potentially salvageable tissue is becoming increasingly important in a real-life setting.

The evaluation of the presumable infarct core volume by means of MR diffusion-weighted imaging (DWI) and the volume of putatively critically hypoperfused brain tissue by means of MR perfusion imaging (PWI) could provide crucial information about potentially salvageable ischemic brain tissue that could be functionally recovered, if perfusion is restored sufficiently. The difference between both volumes (i.e., the assumed penumbra) usually referred to as perfusion-diffusion mismatch represents an approximate measure of the tissue at risk and has been investigated in detail over the last two decades [13, 14]. Besides computed tomography perfusion (CTP), the most commonly used method to access perfusion parameters is by means of dynamic susceptibility contrast MR imaging (DSC-MRI), a method utilizing the signal drop caused by paramagnetic contrast media in T_2_- or T_2_^*^-weighted images. The evaluation of contrast media dynamics involves complex mathematical operations to estimate the volume size of hypoperfused tissue [15]. In DWI-MRI, the infarct core can be delineated by regions of hypointense signal in ADC images due to the cytotoxic edema. The apparent diffusion coefficient (ADC) is usually used to estimate the size of the infarct core. A large ADC core of > 70 ml has been demonstrated to relate to worse clinical outcome [16]. Hence, quantification of the infarct core (with or without perfusion-diffusion mismatch) has been regarded as mandatory by AHA/ASO recommendations in the process of decision-making for MT to select those patients that will most likely benefit from such treatment [12, 17].

Given that many of the steps involved in the evaluation of the perfusion-diffusion mismatch are user dependent and the consistency of the quantitative evaluation highly correlates with user experience, an automatic software support with minimal user interaction is appreciated in clinical routine. Specifically, the volumetric quantification of both hypoperfused tissue and ADC is crucial, as these parameters define the perfusion-diffusion mismatch and, thus, may influence therapeutic decisions. Volumetric quantification can be done manually, which is time consuming and user dependent, or requires additional image processing methods. Because of this need, several software applications for quantitative analysis in acute ischemic stroke have been developed, offering a fully automated perfusion-diffusion mismatch calculation without user interaction to support treatment decisions (e.g., whether a patient receives MT and/or thrombolysis, respectively, or best medical care).

The aim of this study was to compare the performance of two software applications, RAPID® (iSchemaView) and Olea Sphere® (Olea Medical). Perfusion-diffusion mismatch volumes and their dependence from hypoperfused tissue and ADC volumes were explored in this retrospective study. We investigated if suggested limits for therapeutic treatment decisions, recommended by seminal studies, have general validity or should be considered individually for different software systems.

## Methods

### Patients

We analyzed brain MRI examinations of consecutive patients with clinical symptoms of acute stroke and LVO of the anterior circulation between 2012 and 2018 jointly managed or treated by the Department of Neurology and the Division of Neuroradiology, Vascular and Interventional Radiology at the Medical University of Graz. Patients were retrospectively identified using the internal picture archiving and communication system (PACS). We excluded patients not receiving MR perfusion imaging as well as patients without LVO, stroke of the vertebrobasilar circulation, and stroke mimics without detected DWI lesion or vessel occlusion. Eighty-seven patients met those criteria. In six patients, automatic evaluation of perfusion-diffusion mismatch failed either in one or both software applications. A failed calculation was considered when either perfusion data or diffusion data were not provided in one or both of the two software packages due to severe motion artifacts. Finally, a total of 81 patients (38 females, mean age 68.8 ± 12.7 years, range 27–88 years, median NIHSS 11) were included in our analysis. Of these, 53 had occlusions of the middle cerebral artery (M1 or M2 segment), 21 of the internal carotid artery, five of the terminal internal carotid artery, and two patients had tandem occlusions of the internal carotid artery and the middle cerebral artery, respectively.

This study was approved by the local ethics committee in accordance with the declaration of Helsinki.

### Magnetic resonance imaging data acquisition

MRI was performed on a single 1.5-Tesla clinical scanner (SIEMENS MAGNETOM Espree, Siemens Healthineers) according to the standard protocol used at our hospital for the workup of patients with acute stroke. This protocol includes the following sequences: axial diffusion-weighted single-shot echo planar imaging (TR/TE 4000/88 ms; isotropic diffusion weighting; *b* values 0 and 1000 s/mm^2^, matrix 128 × 128, FOV 240 × 240 mm^2^), axial perfusion imaging (TR/TE 1800 ms, 40 ms, slice thickness 5 mm, matrix 128 × 128, FOV 240 × 240 mm^2^, flip angle 60), axial fluid-attenuated inversion recovery (TR/TE 8000 ms/99 ms, slice thickness 5 mm) or optional axial fluid-attenuated inversion recovery blade (TR/TE 8500 ms/98 ms, slice thickness 5 mm), axial T2-weighted (TR/TE 49 ms, 40 ms, slice thickness 2.5 mm), before 2015 axial T2*-weighted GE (TR/TE 936 ms, 26 ms, slice thickness 5 mm), axial 3D TOF angiography (TR/TE 26 ms, 7.00 ms, slice thickness 0.8 mm), axial T1-weighted post contrast media (TR/TE 690 ms, 17 ms, slice thickness 5 mm), and optional angiography of the extra cranial vessels using fast low angle shot magnetic resonance imaging. Contrast media Gadovist (Bayer Vital GmbH), ProHance (Bracco Imaging), and Dotarem (Guerbet) were administered intravenously via a power injector (Spectris; Medrad Inc.) with a dose of 0.1 mmol/kg body weight at 4 ml/s flow followed by 20 ml of NaCl 0.9% at the same flow. ProHance is the standard contrast medium that is substituted by Gadovist or Dotarem in the rare cases of known intolerance. The total acquisition time took 14 min and 18 min, respectively, with the additional extra cranial angiography.

### Automated perfusion-diffusion mismatch calculation

**Fig. 1:**
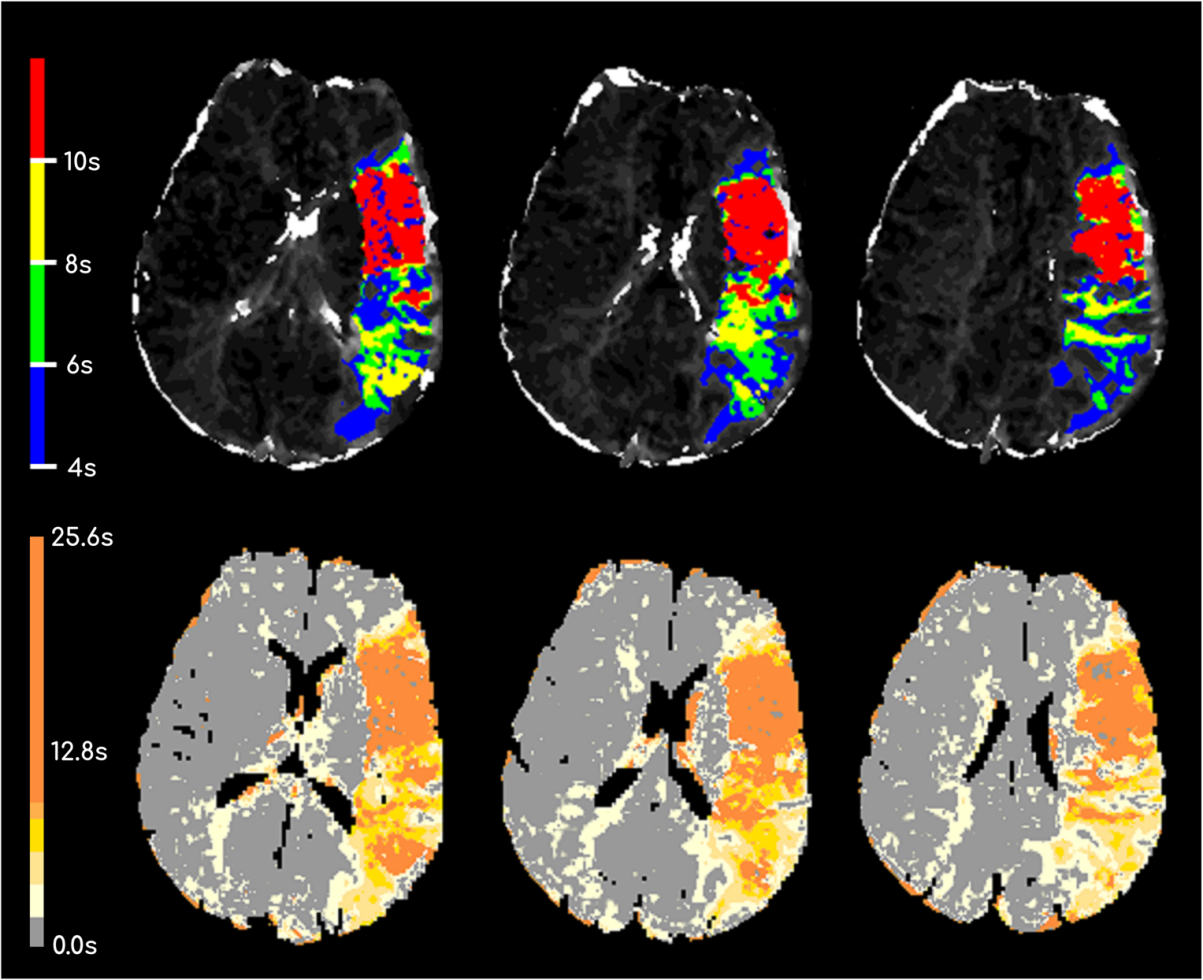
T_max_ maps from central slices of one patient evaluated with RAPID® (first row) and Olea Sphere® (second row). Please note that different colormaps were used by the two software packages to visualize the time to the maximum of the residue function

The RAPID**®** software was installed in our hospital 2012 as prerequisite for participation in the SWIFT PRIME stroke trial [9]. The Olea Sphere**®** DSC plug-in for automated calculation of diffusion-perfusion mismatch was installed 2018 as part of the Olea Sphere**®** post processing package (including volumetric tumor assessment tractography etc.). Both commercially available software packages are used in the daily clinical routine and there is no personal or financial relationship to the vendors. Quantitative automated perfusion-diffusion mismatch calculation performed by RAPID**®** was recalculated with Olea Sphere**®** software for all patients in this study. RAPID**®** and Olea Sphere**®** are two fully automated post processing software applications used for quantitative stroke analysis. Both applications provide perfusion and diffusion maps such as relative cerebral blood flow, relative blood volume, and relative mean transit time. Furthermore, both software applications quantify the volume of hypoperfused tissue and ADC lesion and automatically calculate the volume of perfusion-diffusion mismatch. Decreased perfusion is quantified on T_max_ maps, an estimate of delayed blood supply whereby a delay of 6 s (T_max_ > 6 s) [18, 19] of the time to the maximum of the residue function was chosen as a threshold for hypoperfused tissue for both software applications (see Fig. 1). Threshold for ADC (620 × 10^−6^ mm^2^/s) volume estimation was also identical for both software applications [20]. Perfusion-diffusion mismatch, the penumbra, is defined as the difference in the volume of segmented hypoperfused tissue volume and the segmented volume of the ADC lesion. All volumes are given in milliliter (ml).

All MRI source images along with perfusion-diffusion mismatch calculation summaries from RAPID® and Olea Sphere® were independently reviewed and checked for validity by two experienced neuroradiologists (N.H. and U.W.). The neuroradiologists were not blinded to the software. However, the reports were evaluated in terms of correct placement of the arterial input function, and misregistration of regions outside the territory of the occluded artery, respectively. Moreover, studies with distinctive motion artifacts that rendered the results of the automated segmentation useless had been excluded. The morphological MRIs were assessed for T_2_-FLAIR lesions, hemorrhage, and vessel occlusions.

### Statistical analysis

Statistical analyses were performed using the software SPSS 24 (IBM) and OriginPro 2018 (OriginLab Corporation). For the comparison of parameters evaluated by the two software applications RAPID® and Olea Sphere®, scatterplots were used to investigate the correlation between both methods. Bland-Altman plots were used to detect systematic biases in one of the two methods. For all volume differences in the Bland-Altman plots, we calculated mean ADC volume differences (Volume_RAPID®_–Volume_Olea Sphere®_). Descriptive statistics was used to quantify the volume differences between both software packages for hypoperfused tissue, ADC, and perfusion-diffusion mismatch (mean values for normal distributed values and median values for non-normal distribution). The Shapiro-Wilk test was used to test for normal distribution of data. Not surprisingly, the estimated volumes were not normal distributed since occlusions of different vascular sections lead to a hypoperfusion of certain cerebral areas. A test for significant differences was therefore performed with the Wilcoxon signed-rank test. Results were reported with *z*-score (z) and Pearson’s correlation coefficient (*r*) to report effect size.

## Results

**Fig. 2:**
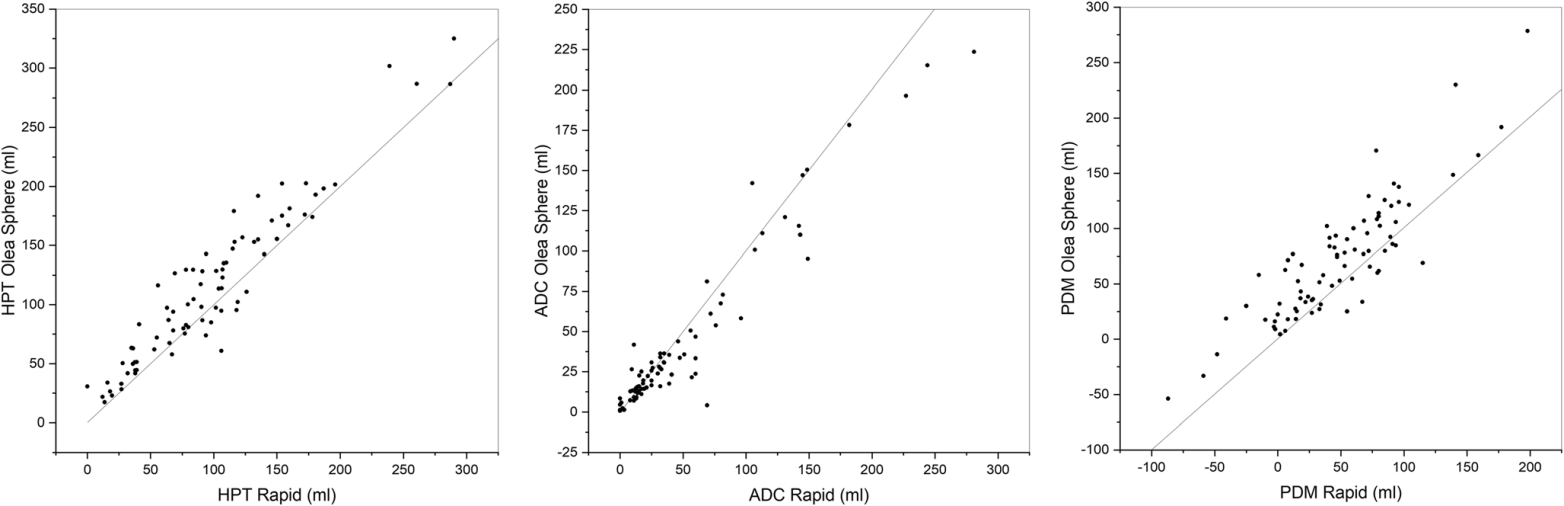
Scatterplots of automatically evaluated volumes of hypoperfused tissue (HPT), apparent diffusion coefficient (ADC), and perfusion-diffusion mismatch (PDM) demonstrating the correlations between the results from RAPID® and Olea Sphere® software

**Fig. 3:**
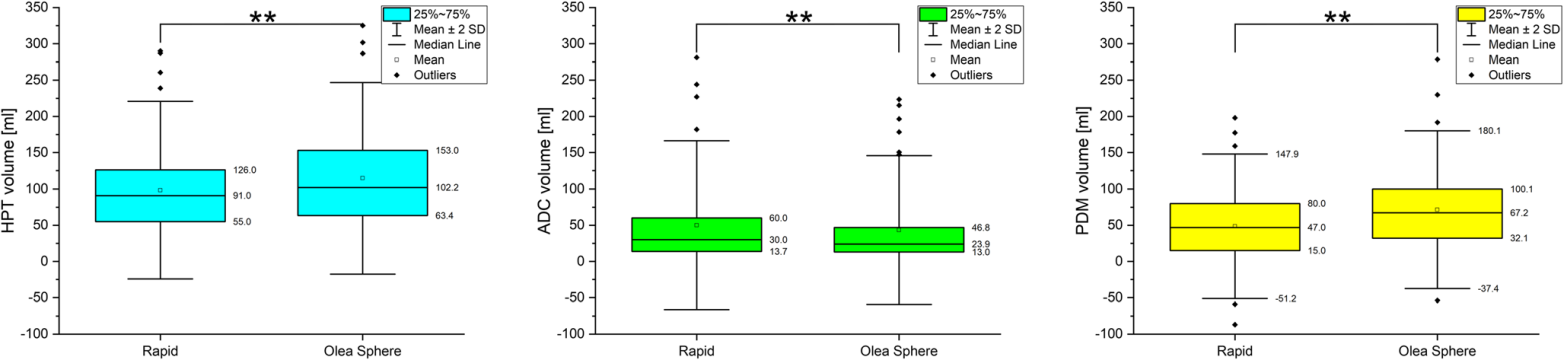
Descriptive statistics of hypoperfused tissue (HPT), apparent diffusion coefficient (ADC), and perfusion-diffusion mismatch (PDM) evaluated automatically with RAPID® and Olea Sphere® software, demonstrating a significant difference in HPT and ADC and a highly significant difference in PDM (*p* < 0.05)

Scatter plots of hypoperfused tissue and ADC tissue volumes showed a good agreement between RAPID**®** and Olea Sphere**®** calculations (Fig. 2). However, the perfusion-diffusion mismatch determined by the two computer programs were significantly different (Fig. 3). The median volume of the perfusion-diffusion mismatch was 47.0 ml for RAPID**®** and 67.2 ml for Olea Sphere**®**, respectively (*p* < 0.05, *z* = − 6.32, *r* = 0.70). The median of volume of hypoperfused tissue was 91.0 ml for RAPID**®** and 102.2 ml for Olea Sphere**®**, respectively (*p* < 0.05, *z* = − 6.03, *r* = 0.67). ADC volume differences were determined as 30.0 ml for RAPID**®** and 23.9 ml for Olea Sphere**®** (*p* < 0.05, *z* = 3.77, *r* = 0.42), respectively (Fig. 3).

**Fig. 4:**
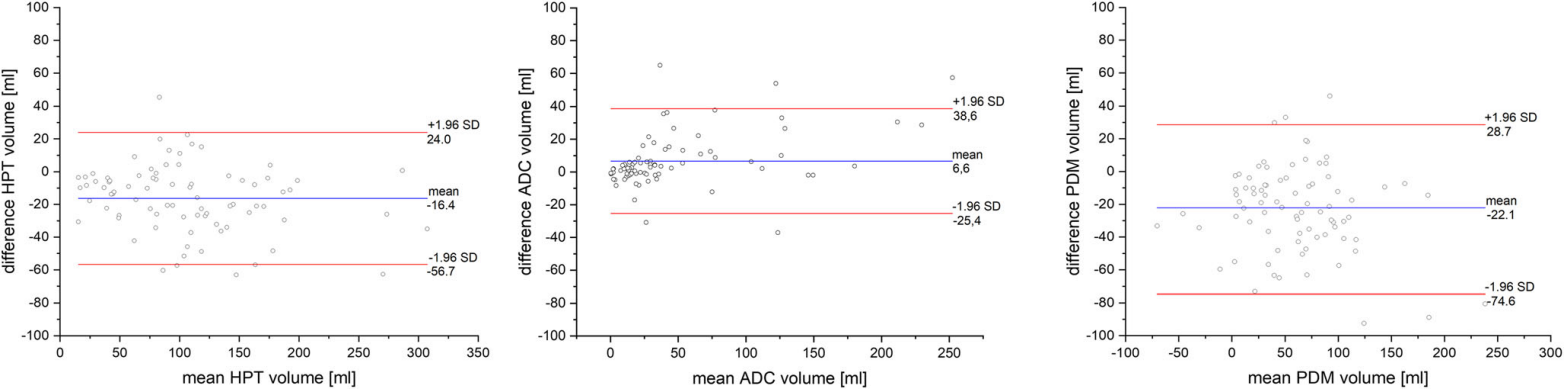
Bland-Altman plots comparing RAPID® and Olea Sphere® software for the volumes of hypoperfused tissue (HPT), apparent diffusion coefficient (ADC), and perfusion-diffusion mismatch (PDM). Volumes were evaluated as Volume_RAPID®_–Volume_Olea Sphere®_

Bland-Altman analyses revealed that the hypoperfused tissue volume was largely estimated with Olea Sphere® compared with RAPID® (hypoperfused tissue mean = − 13.8 ml). ADC lesion volume was slightly largely estimated by RAPID® (ADC mean = 8.2 ml). Perfusion-diffusion mismatch volume was found to be larger when using Olea Sphere® (perfusion-diffusion mismatch mean = − 22.1 ml) (see Fig. 4). These results suggest the presence of a systematic bias when comparing both methods. However, no method over- or underestimated the evaluated mean volumes to a large extent.

**Fig. 5:**
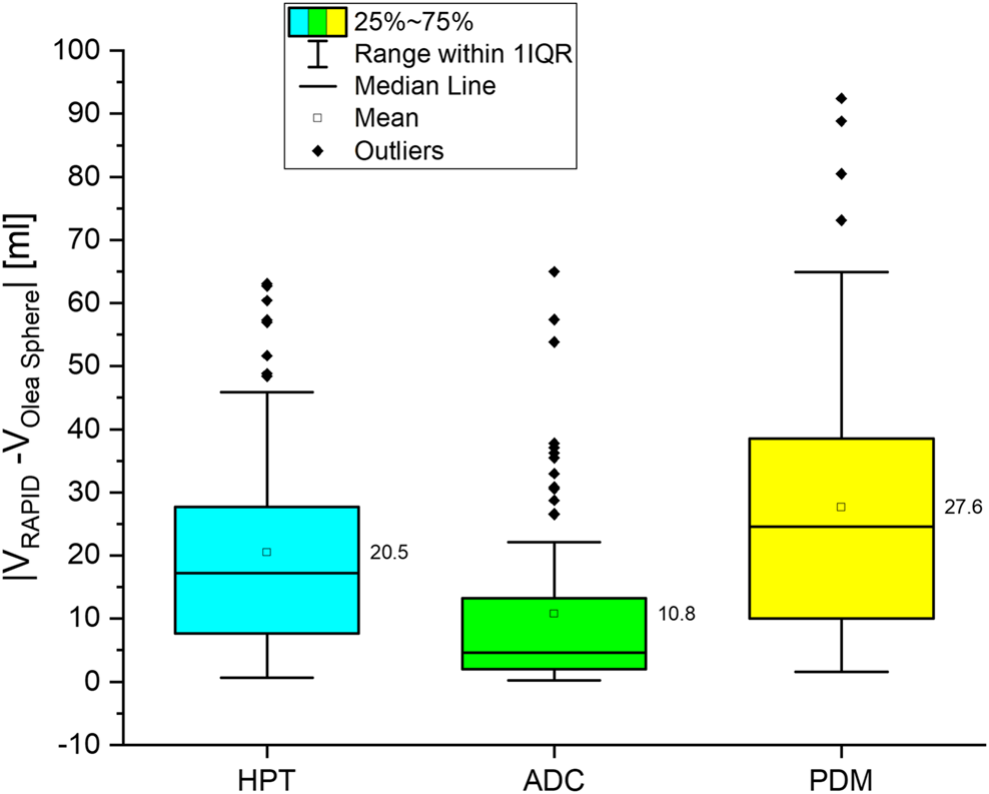
Absolute differences in hypoperfused tissue (HPT), apparent diffusion coefficient (ADC), and perfusion-diffusion mismatch (PDM) between RAPID® software and Olea Sphere® software

For the support of a therapeutic decision, the absolute difference between both methods on a single-subject basis is of utmost interest. The mean absolute difference for hypoperfused tissue volume was 20.5 ml between software packages. A smaller difference between Olea Sphere® and RAPID® was found for ADC volumes (10.8 ml) and the largest difference was found for perfusion-diffusion mismatch volume (27.6 ml) (Fig. 5). Differences between software packages were as large as 63.1 ml for hypoperfused tissue, 65.0 ml for ADC, and 92.4 ml for perfusion-diffusion mismatch volumes, respectively.

**Fig. 6:**
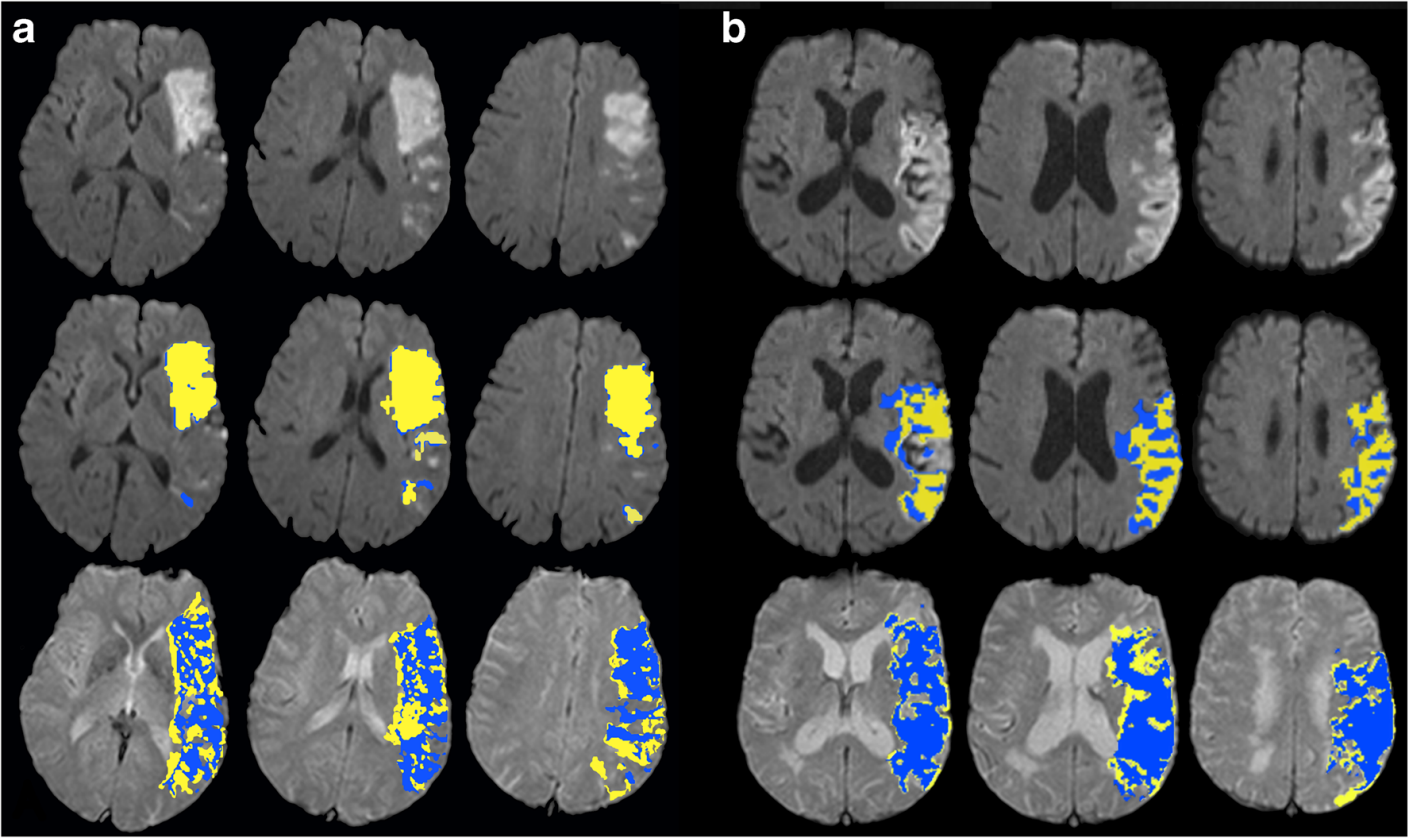
Two patients with outlined apparent diffusion coefficient (ADC) lesion volumes (first row) and hypoperfused tissue volumes (second row) evaluated automatically by RAPID**®** software (blue) and Olea Sphere® software (yellow). Patient **a** shows a good agreement of ADC lesion volumes with only a small difference of 11 ml (18%). A larger difference of 37 ml (41%) was observed for hypoperfused tissue volume. In patient **b**, the difference of the calculated ADC volumes of 43 ml (81%) is large. A better agreement with a difference of 27 ml (25%) was observed for the hypoperfused tissue

Examples for different hypoperfused tissue volumes and ADC volumes, determined by RAPID® (blue regions) and Olea Sphere® (yellow regions), in two exemplary patients are presented in Fig. 6. In patient A, the diffusion volumes are in good agreement (72.0 ml RAPID® vs. 61.1 ml Olea Sphere®) while there is a larger difference in the outlined hypoperfused tissue volumes (91.0 ml RAPID® vs. 128.3 ml Olea Sphere®). Patient B shows a larger difference in ADC volume (96.0 ml RAPID® vs. 58.2 ml Olea Sphere®) and a better agreement in hypoperfused tissue volumes (108.0 ml RAPID® vs. 135.1 ml Olea Sphere®).

After applying a threshold of < 70 ml for the ADC volume of the infarct core that was derived from the inclusion criteria of the randomized multicenter DEFUSE 3 trial [10], 47/81 (58%) patients (Olea Sphere®) and 41/81 (50.6%) patients (RAPID®) would have been deemed eligible for mechanical thrombectomy. Application of the DEFUSE 3 threshold of 70 ml infarction core would have resulted in dissenting treatment decisions in 6/81(7.4%) patients.

## Discussion

Determination of the infarct core volume and the amount of potentially salvageable brain tissue (penumbra) after LVO are important predictive factors for the functional outcome of patients treated with MT [21]. A recent meta-analysis indicated that selection of acute ischemic stroke patients, using advanced neuroimaging methods, is associated with improved clinical outcomes [22]. Therefore, advanced imaging techniques, such as MR-PWI and MR-DWI, that allow for quantifying the volume of the infarct core and penumbra play an increasingly important role in the management of acute stroke [23, 24]. For estimation of hypoperfused tissue volume, ADC lesion volume, and perfusion-diffusion mismatch volume, various software packages have been developed in the last decade. To meet the requirements for a clinically applicable therapy decision support, all manufacturers aim for automated volume estimation with minimal user interaction to ensure operator independence. Recently published thrombectomy trials used RAPID® to determine the volume size of the infarct core and the size of the penumbra [8, 10, 11]. In these trials, patients with an infarct core > 70 ml or an infarct core volume above a specified threshold were deemed unsuitable for MT.

In our study, we compared two established software products (RAPID® and Olea Sphere®) with regard to volume estimation of hypoperfused tissue, ADC, and perfusion-diffusion mismatch. There is a general consistency between both software programs specifically when comparing the mean values of hypoperfused tissue, ADC, and perfusion-diffusion mismatch. A small systematic difference was observed in the sense that RAPID® outlines smaller volumes of hypoperfused tissue but slightly larger ADC volumes. This fact becomes more important when looking at the absolute difference between both methods. An absolute difference between RAPID® and Olea Sphere® of nearly 30 ml perfusion-diffusion mismatch volume suggests that a strict limit of perfusion-diffusion mismatch > 70 ml for the decision whether MT is an appropriate therapy might not be transferable to other software products. Potential reasons for the perfusion-diffusion mismatch volume discrepancies may be the different segmentation algorithm for the hypoperfused tissue and ADC volumes. Although the identified regions for hypoperfused tissue and ADC are congruent, small differences of the outlined regions in single slices can sum up to considerable differences in 3D volumes. However, differences of hypoperfused tissue and ADC volumes estimated with RAPID® and Olea Sphere® seemed to be independent from volume size. This suggests that a constant correction factor might be applied to perfusion-diffusion mismatch volume limits for each software package to ensure comparable results with respect to therapy decision. Our results are in line with the work of Yunyun Xiong et al [25] in that the authors also observed differences in the volume of the infarct core for both software packages. They showed that RAPID® core volumes were larger than Olea Sphere® core volumes if a threshold of rCBF < 30% is applied. However, results are not directly comparable with our results as CT perfusion is based on relative cerebral blood flow (rCBF) to delineate the infarct core.

In our study, 6/81 patients would not have met the inclusion criteria for the DEFUSE 3 trial when the results of RAPID® were taken into account. Assuming the absence of other conflicting factors, strict application of the 70 ml volume limit would have led to the exclusion of these patients from mechanical thrombectomy. However, in this study, only two software packages were investigated. Further studies will be necessary comparing more software packages for automated perfusion-diffusion mismatch evaluation to validate suggested limits for therapy decision on a broader basis.

Several possible limitations must be considered when interpreting the results of our study. Firstly, identification of MRI studies and automated volume calculation was performed retrospectively. However, as all consecutive studies during the time of the investigation were included, the RAPID® and Olea Sphere® processing were performed on the same MRI source images, and no manual correction was performed during the segmentation process; the typical drawbacks of retrospective data acquisition were minimized for our study. Secondly, the compared software packages are applying different segmentation algorithms for calculation of the volume of hypoperfused tissue and ADC. The exact underlying algorithm is not known. As the use of such software in the clinical routine is considered to be without user interaction, the missing in-depth knowledge about the underlying algorithms does not influence the results of our study. Thirdly, we did not attempt to correlate the results with the clinical outcome of the patients or the final stroke volume in the follow-up imaging. However, as the primary goal of the study was to evaluate the technical differences of the two software packages in stroke diagnostics, correlation to the clinical outcome would have meant a research question on its own. Furthermore, additional calculation of the spatial overlap between lesions was not performed. In the automated perfusion-diffusion mismatch evaluation, the user has no access to the outlined regions. Therefore, a post processing of the outlined regions in terms of quantifying the overlap, for example with estimating the Dice coefficient, was not possible. However, two experienced neuroradiologists have examined all 81 patients and confirmed the congruence of hypoperfused tissue and ADC for the two evaluations on a visual basis.

## Conclusion

The benefits of using automated imaging software to support clinical decisions in acute ischemic stroke are undoubted. However, this study revealed that volume segmentation in different software products may lead to significantly different result in the individual patient and may thus seriously influence the decision for or against mechanical thrombectomy. Thresholds used for therapeutic decisions and based on absolute numbers might not be transferable between different software platforms.

## Abbreviations

LVO: Large vessel occlusion
MT: Mechanical thrombectomy
